# Microwave-Assisted Synthesis of SnO_2_@ZnIn_2_S_4_ Composites for Highly Efficient Photocatalytic Hydrogen Evolution

**DOI:** 10.3390/ma17102367

**Published:** 2024-05-15

**Authors:** Yu-Cheng Chang, Jia-Ning Bi, Kuan-Yin Pan, Yung-Chang Chiao

**Affiliations:** 1 Department of Materials Science and Engineering, Feng Chia University, Taichung 40724, Taiwan; stevensandy559@gmail.com (J.-N.B.); tina0976481935@gmail.com (K.-Y.P.); as25874115@gmail.com (Y.-C.C.); 2 Department of Materials Science and Engineering, National Yang Ming Chiao Tung University, Hsinchu 30010, Taiwan

**Keywords:** SnO_2_@ZnIn_2_S_4_ composites, photocatalytic tap water splitting, microwave-assisted synthesis, SnO_2_ nanoparticles, photocatalytic hydrogen production, reusability

## Abstract

This research successfully synthesized SnO_2_@ZnIn_2_S_4_ composites for photocatalytic tap water splitting using a rapid two-step microwave-assisted synthesis method. This study investigated the impact of incorporating a fixed quantity of SnO_2_ nanoparticles and combining them with various materials to form composites, aiming to enhance photocatalytic hydrogen production. Additionally, different weights of SnO_2_ nanoparticles were added to the ZnIn_2_S_4_ reaction precursor to prepare SnO_2_@ZnIn_2_S_4_ composites for photocatalytic hydrogen production. Notably, the photocatalytic efficiency of SnO_2_@ZnIn_2_S_4_ composites is substantially higher than that of pure SnO_2_ nanoparticles and ZnIn_2_S_4_ nanosheets: 17.9-fold and 6.3-fold, respectively. The enhancement is credited to the successful use of visible light and the facilitation of electron transfer across the heterojunction, leading to the efficient dissociation of electron–hole pairs. Additionally, evaluations of recyclability demonstrated the remarkable longevity of SnO_2_@ZnIn_2_S_4_ composites, maintaining high levels of photocatalytic hydrogen production over eight cycles without significant efficiency loss, indicating their impressive durability. This investigation presents a promising strategy for crafting and producing environmentally sustainable SnO_2_@ZnIn_2_S_4_ composites with prospective implementations in photocatalytic hydrogen generation.

## 1. Introduction

Recently, excessive environmental pollution and severe energy shortages have adversely affected human life and health, prompting research into renewable energy sources [1]. Hydrogen, characterized by its zero-carbon emission properties, is cost-effective, sustainable, and environmentally friendly, making it poised to become a crucial energy source shortly [2]. As an efficient and green method, photocatalysis has been extensively studied for energy conversion, utilizing inexhaustible solar energy to address the issues [3]. Through photocatalytic reactions, solar energy is converted into chemical energy using photocatalysts to decompose water and produce hydrogen [4]. Various semiconductor metal oxide photocatalysts have recently emerged, showcasing elevated photocatalytic efficiency, non-hazardous properties, economic feasibility, and robust chemical resilience [5,6]. Among them, semiconductor metal oxide materials like tin dioxide (SnO_2_) have attracted considerable attention because of their outstanding physical and chemical characteristics [7,8,9]. While predominantly employed for the photocatalytic degradation of organic pollutants, their use in the photocatalytic water splitting process for hydrogen generation remains somewhat limited [10].

Tin dioxide (SnO_2_) is classified as an n-type semiconductor material, possessing a wide band gap of approximately 3.6 eV at room temperature [8,11,12]. It is currently regarded as one of the most promising metal oxides due to its non-toxicity, reliable stability, and excellent optoelectronic properties [13]. SnO_2_ has been widely utilized in gas sensors, electrode materials, lithium-ion batteries, solar cells, and photocatalytic degradation of organic and inorganic pollutants [14,15]. It possesses good electron mobility and a large specific surface area, facilitating its use in photocatalysis and electron transfer [16]. However, its relatively wide bandgap exhibits optimal photocatalytic activity only in ultraviolet, with reduced performance under visible light [17]. Therefore, incorporating other materials or doping other metals is necessary to enhance SnO_2_’s absorption of visible light and consequently improve its visible light photocatalytic activity [18,19,20].

Zinc indium sulfide (ZnIn_2_S_4_) is a ternary metal sulfide known for its layered structure, exhibiting three different crystal phases: cubic, hexagonal, and rhombohedral [21,22,23,24]. This photocatalyst is considered a conventional material for visible light absorption and is categorized as a direct band gap semiconductor with adjustable band gaps ranging from 2.06 to 2.85 eV [23,25,26]. ZnIn_2_S_4_ exhibits minimal toxicity, straightforward synthesis, and exceptional chemical durability, rendering it extensively investigated in areas such as photocatalysis, thermoelectricity, and electrochemical energy storage [27,28]. However, the material’s ability to catalyze through light exposure is limited by quick recombination rates of electron–hole pairs, low mobility of charge carriers, and structural flaws [29,30]. To address these limitations, elemental doping and heterojunction construction increase catalytic active sites and enhance photocatalytic reaction rates [31,32,33]. In previous studies, the combination of SnO_2_ and ZnIn_2_S_4_ has not been explored for the photocatalytic decomposition of tap water to produce hydrogen. This study addresses the respective limitations of SnO_2_ and ZnIn_2_S_4_ by combining them to form SnO_2_@ZnIn_2_S_4_ composites for enhancing their photocatalytic tap water splitting. The composites showed significantly higher efficiency than pure SnO_2_ nanoparticles or ZnIn_2_S_4_ nanosheets, credited to visible light utilization and improved electron transfer across the heterojunction, leading to efficient separation of electron–hole pairs. Additionally, the composites demonstrated remarkable recyclability, maintaining high levels of photocatalytic hydrogen production over eight cycles without significant efficiency loss, suggesting their impressive durability and potential for environmentally sustainable applications in photocatalytic hydrogen generation.

## 2. Materials and Methods

### 2.1. Chemicals

All chemical reagents were procured from commercial suppliers and employed without additional purification processes. Tin(IV) Chloride Pentahydrate (SnCl_4_, 98%, Alfa Aesar, Ward Hill, MA, USA), sodium hydroxide (NaOH, 96%, Showa, Osaka, Japan), citric acid anhydrous (99%, Showa, Osaka, Japan), indium (III) chloride anhydrous (InCl_3_, 98%, Alfa Aesar, Ward Hill, MA, USA), zinc chloride anhydrous (ZnCl_2_, 98%, Alfa Aesar, Ward Hill, MA, USA), thioacetamide (TAA, 98%, Alfa Aesar, Ward Hill, MA, USA), ethanol (99.8%, Sigma-Aldrich, Darmstadt, Germany), methanol (99.8%, Sigma-Aldrich, Darmstadt, Germany), formic acid (96%, Alfa Aesar, Ward Hill, MA, USA), sodium sulfide nonahydrate (Na_2_S, 98%, Alfa Aesar, Ward Hill, MA, USA), sodium sulfite anhydrous (Na_2_SO_3_, 98%, Alfa Aesar, Ward Hill, MA, USA) were utilized in these trials. Deionized water with a higher resistivity greater than 18.2 MΩ prepared all reaction solutions.

### 2.2. Fabrication of SnO_2_ Nanoparticles

An adapted protocol from the existing literature was employed to synthesize SnO_2_ nanoparticles utilizing a microwave-assisted synthesis approach [16]. A total of 0.45 g of Tin(IV) chloride, 0.1 g of citric acid, and 0.4 g of sodium hydroxide were dissolved in 50 mL of deionized water and subjected to ultrasonic vibration for approximately 20 min to ensure homogeneous mixing. Subsequently, the solution was transferred to a 100 mL Teflon reaction vessel and subjected to microwave-assisted hydrothermal heating at 180 °C for 60 min. Upon cooling the reaction to ambient temperature, the solution underwent multiple washes with deionized water and ethanol, followed by centrifugation for 3 min, and ultimately dried in a 70 °C oven to produce the white powder.

### 2.3. Fabrication of SnO_2_@ZnIn_2_S_4_ Composites

The SnO_2_@ZnIn_2_S_4_ composites were synthesized using a microwave-assisted synthesis method. Different weights of SnO_2_ nanoparticles were mixed uniformly with zinc chloride (1.25 mM), indium chloride (2.5 mM), and TAA (5 mM) in a ratio of 1:3 with ethanol and de-ionized water, treated with ultrasonication, transferred into 100 mL Teflon reaction bottle, and underwent a microwave-assisted hydrothermal method to heat at 180 °C for 60 min. Once the reaction concluded and cooled to ambient temperature, the solution underwent rinsing with deionized water and ethanol, followed by centrifugation for 3 min, and subsequently dried in a 70 °C oven to yield the yellow powder of the composite material.

### 2.4. Characterization

The overall crystal structure of the SnO_2_ nanoparticles and SnO_2_@ZnIn_2_S_4_ composites was analyzed using X-ray diffraction (XRD) at a facility in Billerica, MA, USA, utilizing a Bruker D2 phaser system with Cu Kα radiation (λ = 1.5418 Å). The surface characteristics of the SnO_2_ nanoparticles and SnO_2_@ZnIn_2_S_4_ composites were examined through field-emission scanning electron microscopy (FESEM) in Tokyo, Japan, using a Hitachi S-4800 microscope operating at a 15 kV accelerating voltage. Field-emission transmission electron microscopy (FETEM) at 200 kV was employed to study the microstructures and composition of the SnO_2_@ZnIn_2_S_4_ composites with a JEOL 2100F microscope in Tokyo, Japan. The surface chemical composition of the SnO_2_ nanoparticles and SnO_2_@ZnIn_2_S_4_ composites was investigated using X-ray photoelectron spectroscopy (XPS) at a facility in Chigasaki, Japan, equipped with an Al K source. The newly synthesized photocatalysts’ diffused reflectance spectra were analyzed with a UV–visible spectrophotometer (PerkinElmer Lambda 650 S, Waltham, MA, USA). Furthermore, a spectrofluorophotometer obtained room-temperature photoluminescence spectra (Shimadzu, RF-5301PC, Kyoto, Japan). The qualitative analysis of tap water compositions can be conducted using an Inductively Coupled Plasma Optima Optical Emission Spectrometer (ICP-OES, PerkinElmer, OPTIMA 2000DV, Waltham, MA, USA). The components present in tap water include magnesium (Mg), nickel (Ni), barium (Ba), calcium (Ca), cesium (Cs), copper (Cu), iron (Fe), potassium (K), lithium (Li), manganese (Mn), sodium (Na), strontium (Sr), iridium (Ir), boron (B), zinc (Zn), silicon (Si), and tungsten (W).

### 2.5. Photocatalytic Hydrogen Production Experiment

A multi-port reaction system produced hydrogen through photocatalysis with specially made photocatalysts. This setup involved stirring with a magnetic stirrer and a 5 W blue LED light source with a peak wavelength of 420 nm powered via PCX50 B Discover with Perfect Light Technology from Beijing, China. In accordance with the standard procedure, 25 mg of the photocatalysts were introduced into a solution comprising 0.1 M of different sacrificial agents (including Na_2_S, Na_2_SO_3_, CH_2_O_2_, and CH_3_OH) and 50 mL of deionized water. When using 0.1 M Na_2_S as the sacrificial reagent in deionized or tap water, the pH values were 13.0 and 12.9, respectively. Before the commencement of the experiment, a degassing process was conducted for a duration of 10 min to eliminate air from the system. Subsequently, the generated hydrogen was measured utilizing gas chromatography (GC, Shimadzu GC-2014, Kyoto, Japan) equipped with a thermal conductivity detector (TCD).

## 3. Results and Discussion

### Characterization of SnO_2_@ZnIn_2_S_4_ Composites

**Figure 1:**
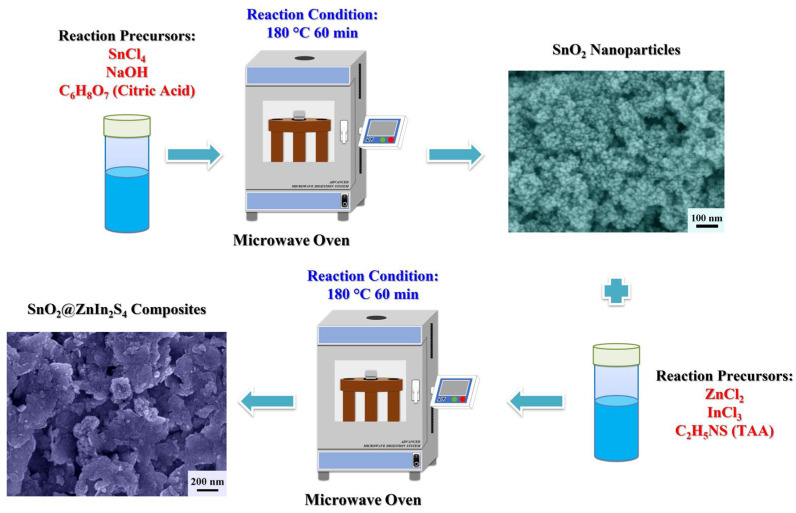
Illustrates a schematic representation of the reaction to forming the SnO_2_@ZnIn_2_S_4_ composites.

Figure 1 illustrates the reaction process of SnO_2_@ZnIn_2_S_4_ composites using a two-step microwave-assisted hydrothermal method. Firstly, SnO_2_ nanoparticles were prepared by microwave-assisted hydrothermal treatment of SnO_2_ precursor (SnCl_4_, NaOH, and citric acid) at 180 °C for 60 min. Subsequently, varying amounts of SnO_2_ nanoparticles were dispersed in the precursor solution of ZnIn_2_S_4_ (ZnCl_2_, InCl_3_, and TAA), followed by another round of microwave-assisted hydrothermal treatment at 180 °C for 60 min to obtain the SnO_2_@ZnIn_2_S_4_ composites.

**Figure 2:**
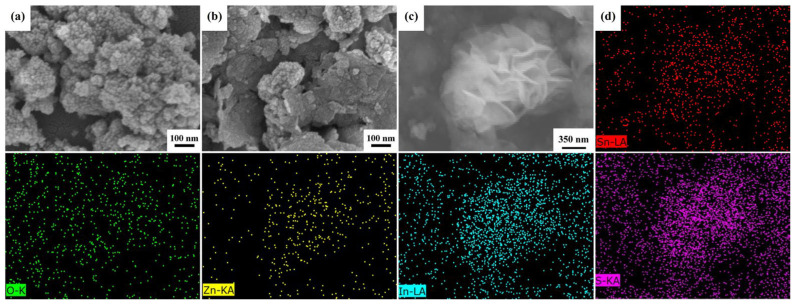
The FESEM images of (**a**) SnO_2_ nanoparticles and (**b**,**c**) SnO_2_@ZnIn_2_S_4_ composites. (**d**) The FESEM EDS mapping images of SnO_2_@ZnIn_2_S_4_ composites.

The morphology of the SnO_2_ nanoparticles and SnO_2_@ZnIn_2_S_4_ composites synthesized using the two-step microwave-assisted hydrothermal method was examined using an FESEM, as shown in Figure 2. The SnO_2_ nanostructures are depicted, comprising numerous nanoparticles with a diameter of approximately 20 nm, uniformly dispersed and aggregated, as shown in Figure 2a. Figure 2b displays the FESEM image of the SnO_2_@ZnIn_2_S_4_ composites following the incorporation of ZnIn_2_S_4_. Notably, a multitude of nanoparticles is observed covering the nanosheet structure. Furthermore, flower-like structures with a similar laminar stacking arrangement can be observed in the low-magnification FESEM image (Figure 2c). Further analysis of the distribution of individual elements through FESEM-EDS mapping is illustrated in Figure 2d. It can be observed from the figures that the SnO_2_@ZnIn_2_S_4_ composites contain Sn, O, Zn, In, and S, and they are evenly distributed. Therefore, it can be demonstrated that SnO_2_ and ZnIn_2_S_4_ successfully combine to form SnO_2_@ZnIn_2_S_4_ composites.

**Figure 3:**
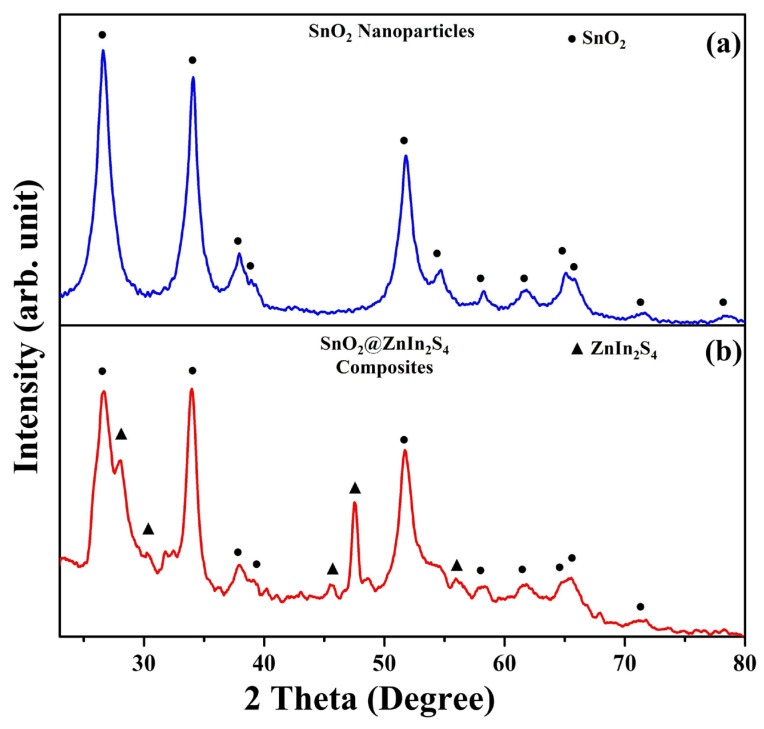
The XRD pattern of (**a**) SnO_2_ nanoparticles and (**b**) SnO_2_@ZnIn_2_S_4_ composites.

To further analyze the overall changes in crystal structure, X-ray diffraction spectroscopy (XRD) was utilized. The XRD analysis shows the crystalline structure of SnO_2_ nanoparticles and SnO_2_@ZnIn_2_S_4_ composites, as shown in Figure 3. Figure 3a presents the XRD pattern of SnO_2_ nanoparticles, with diffraction peak angles at 26.6°, 33.9°, 38.0°, 39.0°, 51.8°, 54.7°, 58.1°, 61.8°, 64.9°, 65.8°, 71.4°, and 78.6°, corresponding to the crystallographic planes (110), (101), (200), (111), (211), (220), (002), (310), (112), (301), (202), and (321), respectively, as compared to PDF# 01-075-2893. This crystal phase structure indicates a pure tetragonal structure of SnO_2_. Figure 3b shows the XRD diffraction pattern of the SnO_2_@ZnIn_2_S_4_ composites. Apart from the SnO_2_ diffraction peaks, the diffraction peaks are observed at 28.0°, 30.3°, 45.6°, 47.6°, and 56.2°, corresponding to the crystallographic planes (011), (012), (105), (111), and (114), respectively, as compared to PDF# 04-009-4783. This crystal phase structure indicates a hexagonal structure of ZnIn_2_S_4_. Additionally, no other impurity phases were detected in the XRD pattern. This result serves to confirm the successful generation of SnO_2_@ZnIn_2_S_4_ composites.

**Figure 4:**
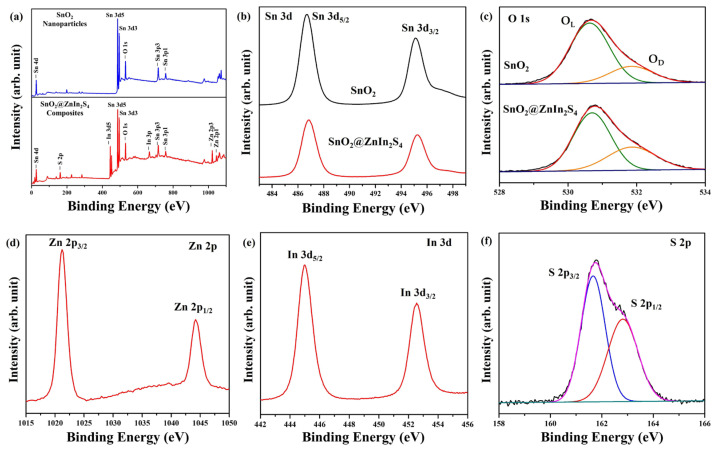
(**a**) The survey XPS spectra of the SnO_2_ nanoparticles and SnO_2_@ZnIn_2_S_4_ composites. High-resolution XPS spectra of (**b**) Sn 3d and (**c**) O 1s for SnO_2_ nanoparticles and SnO_2_@ZnIn_2_S_4_ composites, respectively. High-resolution XPS spectra of (**d**) Zn 2p, (**e**) In 3d, and (**f**) S 2p for SnO_2_@ZnIn_2_S_4_ composites, respectively.

XPS was utilized to examine the chemical compositions of SnO_2_ nanoparticles and SnO_2_@ZnIn_2_S_4_ composites [34]. The XPS survey spectra of the prepared samples, as shown in Figure 4a, distinctly confirm the binding energies associated with C 1s, Sn 3P, Sn 3d, Sn 4d, and O 1s in both SnO_2_ nanoparticles and SnO_2_@ZnIn_2_S_4_ composites. Therefore, Zn, In, and S binding energy characteristics are used to identify SnO_2_ nanoparticles and SnO_2_@ZnIn_2_S_4_ composites. The binding energy for Sn is observed in the SnO_2_ nanoparticles and SnO_2_@ZnIn_2_S_4_ composites at 486.7 eV and 495.1 eV, respectively, as depicted in Figure 4b. These peaks are identified as the Sn 3d_5/2_ and Sn 3d_3/2_ peaks of SnO_2_ [35]. The O 1s binding energy of the SnO_2_ nanoparticles and SnO_2_@ZnIn_2_S_4_ composites is analyzed in Figure 4c. It is separated into two peaks with binding energies at 530.6 eV and 531.8 eV, representing lattice oxygen (O lattice, O_L_) and oxygen-deficient region (O defect, O_D_) in SnO_2_, respectively [36,37]. This outcome shows many oxygen defects in the SnO_2_ nanoparticles and SnO_2_@ZnIn_2_S_4_ composites. The Zn 2p spectrum displays two distinct peaks at 1021.3 eV and 1044.3 eV, corresponding to the Zn 2p_3/2_ and Zn 2p_1/2_ states, respectively, as illustrated in Figure 4d [32]. The In 3d spectrum (Figure 4e) reveals peaks at 445.0 eV and 452.5 eV, which are associated with the In 3d_5/2_ and In 3d_3/2_ states of ZnIn_2_S_4_. These findings align with existing literature [38]. The S 2p spectrum (Figure 4f) exhibits two distinct peaks located at 161.6 eV and 162.8 eV, which have been identified as corresponding to the S 2p_3/2_ and S 2p_1/2_ states, respectively [39]. These results collectively confirm the presence of Zn^2+^, In^3+^, and S^2−^ chemical states, with no indications of impurity peaks in the spectra.

**Figure 5:**
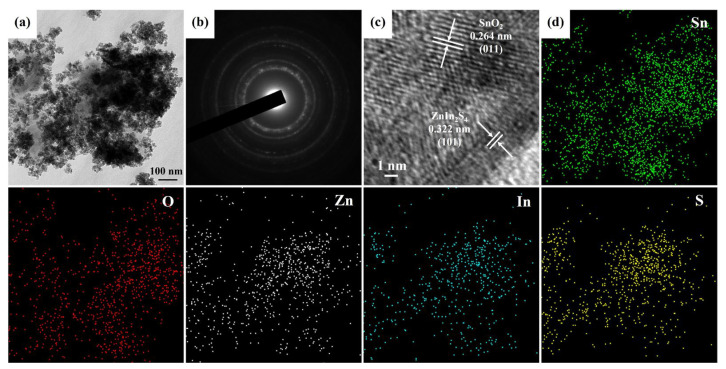
The (**a**) FETEM image, (**b**) SAED pattern, (**c**) HRTEM image, and (**d**) FETEM EDS mapping images of SnO_2_@ZnIn_2_S_4_ composites.

TEM analysis was performed to examine the morphology and crystal structure of the SnO_2_@ZnIn_2_S_4_ composites more efficiently. Figure 5a displays the TEM image, revealing the complete integration of SnO_2_ nanoparticles with ZnIn_2_S_4_ nanosheets. Figure 5b exhibits the SAED pattern of SnO_2_@ZnIn_2_S_4_ composites, showing a polycrystalline ring. The crystal structures consist of tetragonal SnO_2_ (PDF# 01-075-2893) and hexagonal ZnIn_2_S_4_ (PDF# 04-009-4783). Moreover, upon examination using high-resolution transmission electron microscopy (HRTEM) imaging (Figure 5c), lattice spacings of 0.264 nm indicative of the (011) plane of SnO_2_ (PDF# 01-075-2893) and 0.322 nm corresponding to the (101) plane of ZnIn_2_S_4_ (PDF# 04-009-4783) were identified. This observation serves to validate the crystal structures of SnO_2_ and ZnIn_2_S_4_. TEM-EDS mapping image (Figure 5d) was performed for qualitative and semi-quantitative compositional analysis of SnO_2_@ZnIn_2_S_4_ composites, revealing an even distribution of the individual elements Sn, O, Zn, In, and S within the composites. This outcome confirms the effective production of composite materials consisting of SnO_2_ and ZnIn_2_S_4_.

**Figure 6:**
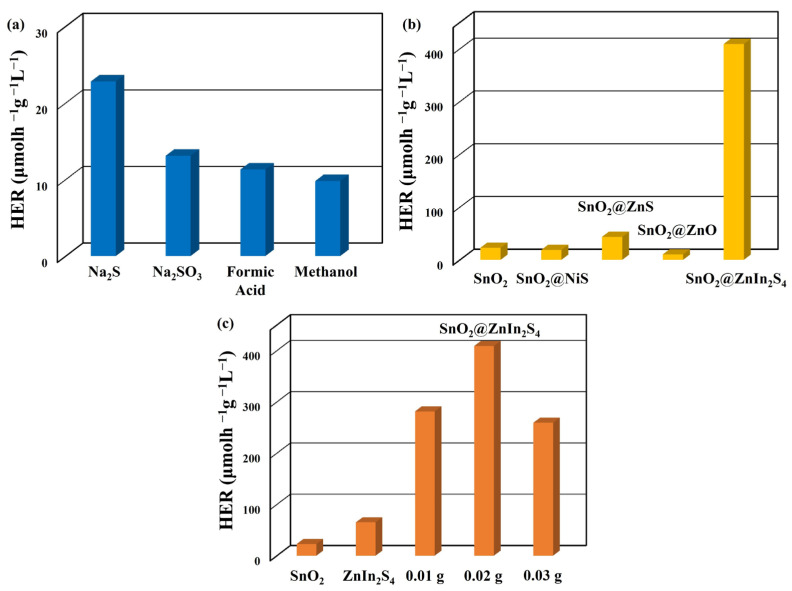
(**a**) The average HER of SnO_2_ nanoparticles with different sacrificial reagents. (**b**) The average HER of SnO_2_ nanoparticles is decorated with various materials. (**c**) The average HER of SnO_2_@ZnIn_2_S_4_ composites with varying weights of SnO_2_ nanoparticles.

The photocatalytic efficiency of the fabricated SnO_2_ nanoparticles was assessed by quantifying the hydrogen evolution rate (HER) in 50 mL deionized water under blue LED light irradiation. In aqueous settings, sacrificial agents are commonly employed to boost the effectiveness of oxidation reactions in the photocatalytic water splitting process for hydrogen production [40]. Figure 6a illustrates the impact of four sacrificial agents (Na_2_S, Na_2_SO_3_, CH_2_O_2_, and CH_3_OH) on the photocatalytic performance of SnO_2_ nanoparticles without pH adjustment. All sacrificial agents were utilized at a uniform concentration of 0.1 M. The average hydrogen evolution rate (HER) with SnO_2_ nanoparticles follows the order: Na_2_S (22.8 μmol·h^−1^·g^−1^·L^−1^), Na_2_SO_3_ (13.1 μmol·h^−1^·g^−1^·L^−1^), CH_2_O_2_ (11.3 μmol·h^−1^·g^−1^·L^−1^), and CH_3_OH (9.8 μmol·h^−1^·g^−1^·L^−1^). A possible rationale behind this phenomenon could be the adsorption of sulfide ions (S^2−^, originating from dissociated Na_2_S) onto the photocatalyst. These ions could interact with photogenerated positive charges, thereby impeding the recombination of electron–hole pairs [41]. Therefore, Na_2_S was used as a sacrificial reagent in the subsequent reactions.

To further boost the effectiveness of photocatalytic water splitting for hydrogen production using SnO_2_ nanoparticles, this investigation also integrated 0.02 g of SnO_2_ nanoparticles into different precursor materials before the reaction. Subsequently, the efficacy of photocatalytic water splitting for hydrogen generation using SnO_2_ nanoparticles in conjunction with various materials was examined under blue LED light exposure, as depicted in Figure 6b. The optimal efficiency for photocatalytic water splitting for hydrogen production (408.9 μmol·h^−1^·g^−1^·L^−1^) is achieved when SnO_2_ nanoparticles react with ZnIn_2_S_4_ precursor materials to form SnO_2_@ZnIn_2_S_4_ composites. This result indicates a 17.9-fold increase in efficiency compared to SnO_2_ nanoparticles alone, demonstrating that the composite with ZnIn_2_S_4_ effectively enhances the photocatalytic efficiency of the material.

Figure 6c illustrates the performance variation in photocatalytic water splitting to produce hydrogen for pure SnO_2_ nanoparticles, ZnIn_2_S_4_ nanosheets, and SnO_2_@ZnIn_2_S_4_ composites formed by reacting different weights of SnO_2_ nanoparticles with ZnIn_2_S_4_ reaction precursors under blue LED light irradiation. Adding 0.02 g of SnO_2_ nanoparticles exhibits the optimal photocatalytic water splitting efficiency for hydrogen production (408.9 μmol·h^−1^·g^−1^·L^−1^). This result represents a significant enhancement compared to pure SnO_2_ nanoparticles (22.8 μmol·h^−1^·g^−1^·L^−1^) and ZnIn_2_S_4_ nanosheets (65.1 μmol·h^−1^·g^−1^·L^−1^) by approximately 17.9-fold and 6.3-fold, respectively. This finding verifies that incorporating an appropriate weight of SnO_2_ nanoparticles assists in preparing SnO_2_@ZnIn_2_S_4_ composites with optimal hydrogen production efficiency.

**Figure 7:**
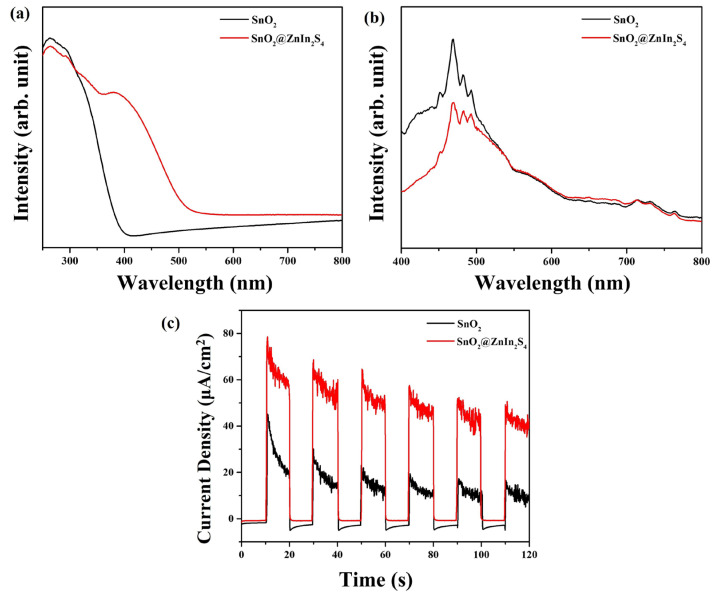
(**a**) UV–visible absorption spectra, (**b**) PL spectra, and (**c**) photocurrent response of SnO_2_ nanoparticles and SnO_2_@ZnIn_2_S_4_ composites.

Figure 7a displays the UV–visible absorption spectra of SnO_2_ nanoparticles and SnO_2_@ZnIn_2_S_4_ composites. In contrast to SnO_2_ nanoparticles, the absorption edge of SnO_2_@ZnIn_2_S_4_ composites demonstrates a significant redshift. Moreover, the absorption intensity of SnO_2_@ZnIn_2_S_4_ composites is notably enhanced in the wavelength range of λ > 450 nm, which augments the generation of photogenerated carriers and enhances photocatalytic performance under visible light. Photoluminescence (PL) spectroscopy is a technique that utilizes the fluorescence produced when unbound charge carriers recombine, providing valuable information on the movement, transfer, and separation of electron–hole pairs generated by light in semiconductor materials [6,42]. Figure 7b reveals the measured PL emission spectra of SnO_2_ nanoparticles and SnO_2_@ZnIn_2_S_4_ composites. SnO_2_@ZnIn_2_S_4_ composites exhibit significantly lower emission intensity than SnO_2_ nanoparticles, indicating suppression of photogenerated hole-electron recombination in the composites. Photocurrent measurements are conducted to evaluate the efficiency of photogenerated electron–hole pair separation for photocatalytic performance. As depicted in Figure 7c, the photocurrent intensity of SnO_2_@ZnIn_2_S_4_ composites exceeds that of SnO_2_ nanoparticles, demonstrating the synergistic effect between SnO_2_ and ZnIn_2_S_4_ in suppressing photogenerated electron–hole recombination.

**Figure 8:**
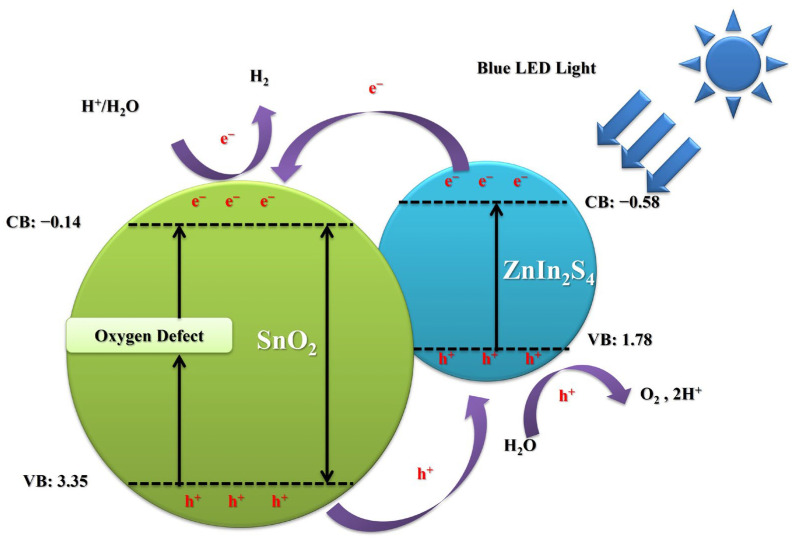
The schematic diagram delineates the photocatalytic mechanism of SnO_2_@ZnIn_2_S_4_ composites.

Figure 8 illustrates the potential mechanism underlying the photocatalytic water-splitting process on the SnO_2_@ZnIn_2_S_4_ composites when subjected to blue LED light irradiation. An ion exchange resin is first applied to the indium tin oxide (ITO) glass, creating a coating of SnO_2_ and ZnIn_2_S_4_. Subsequently, the flat band potential is determined using cyclic voltammetry [43]. The determined valence band (VB) and conduction band (CB) positions of SnO_2_ and ZnIn_2_S_4_ align with established findings. In particular, the conduction band positions for SnO_2_ and ZnIn_2_S_4_ are recorded at −0.14 eV and −0.58 eV, respectively, while their valence band positions are noted at 3.35 eV and 1.78 eV, respectively. [15,44,45,46]. In addition, previous XPS O1s (Figure 4c) can verify that SnO_2_ nanoparticles and SnO_2_@ZnIn_2_S_4_ composites exhibited oxygen defects. Notably, the presence of oxygen defects leads to the emergence of new electronic state bands near the lower boundary of the CB in SnO_2_. This phenomenon reduces the band gap, enhancing visible light absorption [47]. Upon exposure to blue LED light irradiation, photogenerated electrons within the VB of SnO_2_ and ZnIn_2_S_4_ are excited to the CB. Subsequently, the photogenerated electrons in the CB of ZnIn_2_S_4_ can migrate to the CB of SnO_2_. SnO_2_ acts as an electron sink, effectively capturing and reducing hydrogen ions to generate hydrogen. Concurrently, the photogenerated holes within the VB of SnO_2_ can transfer to ZnIn_2_S_4_, where they participate in water oxidation to produce oxygen or hydrogen ions. This photocatalytic process facilitates the efficient separation of photogenerated charge carriers, thereby promoting the photocatalytic production of hydrogen. The collaborative influence notably mitigates carrier recombination, consequently amplifying the photocatalytic efficacy of SnO_2_@ZnIn_2_S_4_ and facilitating effective hydrogen generation. This synergy of photocatalysis helps to separate charge carriers, promoting the efficient production of hydrogen efficiently. Additionally, the combination of SnO_2_@ZnIn_2_S_4_ composites dramatically enhances the ability to absorb light, thereby increasing the effectiveness of generating hydrogen through photocatalysis.

**Figure 9:**
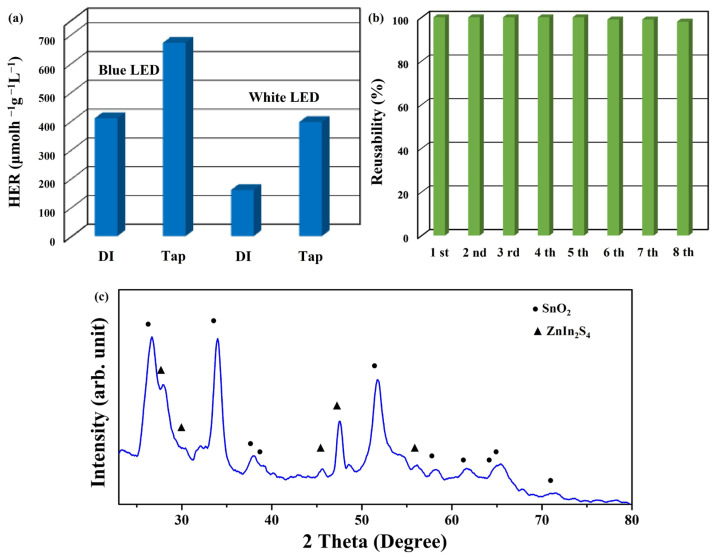
(**a**) The average HER of SnO_2_@ZnIn_2_S_4_ composites under deionized water and tap water under blue or white LED light irradiation. (**b**) Reusability of SnO_2_@ZnIn_2_S_4_ composites for eight cycles. (**c**) XRD spectrum of SnO_2_@ZnIn_2_S_4_ composites after the eight cycles.

Deionized water undergoes purification by eliminating diverse ions and contaminants from tap water [46,48]. Hence, if photocatalysts could directly generate hydrogen via the decomposition of tap water, it could significantly decrease the expenses and duration associated with purifying tap water into deionized water, thereby facilitating the practical utilization of photocatalysts [42]. Additionally, this approach could lead to more sustainable and cost-effective water treatment methods, contributing to environmental conservation efforts [49]. To demonstrate the practicality of utilizing SnO_2_@ZnIn_2_S_4_ composites for photocatalytic tap water splitting, 50 mg of the composites was dispersed in 50 mL of tap water solution containing 0.1 M Na_2_S without any pH modification. This experimental setup was subjected to blue or white LED light irradiation, as depicted in Figure 9a. The average HER of SnO_2_@ZnIn_2_S_4_ composites is 408.9 μmolh^−1^g^−1^L^−1^ in deionized water and 670.8 μmolh^−1^g^−1^L^−1^ in tap water under blue LED light irradiation. The average HER of SnO_2_@ZnIn_2_S_4_ composites is 160.1 μmolh^−1^g^−1^L^−1^ in deionized water and 395.8 μmolh^−1^g^−1^L^−1^ in tap water under white LED light irradiation. Notably, the average HER of SnO_2_@ZnIn_2_S_4_ composites in tap water is approximately 1.64 (blue LED light) and 2.47 (white LED light) times higher than in deionized water, respectively. It is speculated that the possible reason is that more ions or chlorine ions in tap water can facilitate the transfer of photogenerated electron and hole pairs, thereby enhancing photocatalytic hydrogen production [50]. These findings highlight the exceptional photocatalytic efficacy of SnO_2_@ZnIn_2_S_4_ composites in hydrogen generation, even when dealing with intricate water compositions, without needing pH adjustments. In addition, this result also confirms that blue LED light is more effective than white LED light when employing SnO_2_@ZnIn_2_S_4_ composites for the photocatalytic decomposition of tap water to produce hydrogen. The lower photocatalytic efficiency of white LED light compared to blue LED light may be due to the dispersion of light source intensity [41].

Efficiently recycling catalysts is paramount in photocatalysis [41]. Designing photocatalysts that maintain consistent photoactivity across multiple cycles is crucial for minimizing waste and ensuring sustainable processes [51]. In the context of photocatalysts, the gradual decline in efficiency over time can lead to increased waste generation throughout their life cycle. Hence, creating photocatalysts that can be easily separated and recycled is crucial to mitigate the depletion of valuable resources within the waste stream [32]. This study conducted eight consecutive cycles of photocatalytic tap water splitting to assess the durability of SnO_2_@ZnIn_2_S_4_ composites. The average HER of the SnO_2_@ZnIn_2_S_4_ composites exhibits a sustained high level throughout eight cycles, as depicted in Figure 9b. Additionally, the XRD pattern of the sample after the eight cycles, shown in Figure 9c, does not exhibit any new peaks, indicating the preservation of the composite’s structural integrity. These results prove that the SnO_2_@ZnIn_2_S_4_ composites exhibit better durability and recyclability.

## 4. Conclusions

In this study, we have successfully produced composite materials consisting of SnO_2_@ZnIn_2_S_4_ for photocatalytic splitting of tap water, employing a two-step synthesis method assisted by microwave technology. Our investigation delved into the impact of incorporating fixed quantities of SnO_2_ nanoparticles into various materials to form composites, thereby enhancing hydrogen production through photocatalysis. Furthermore, we investigated the effect of different concentrations of SnO_2_ nanoparticles in the ZnIn_2_S_4_ reaction precursor to improve the synthesis of SnO_2_@ZnIn_2_S_4_ composites for photocatalytic hydrogen generation. Notably, the efficiency of photocatalytic hydrogen production in SnO_2_@ZnIn_2_S_4_ composites surpasses that of pure SnO_2_ nanoparticles or ZnIn_2_S_4_ nanosheets. The improved efficiency can be credited to successfully harnessing visible light and promoting photogenerated electrons across the heterojunction, leading to the effective dissociation of electron–hole pairs. Furthermore, reusability tests have validated the exceptional performance of the SnO_2_@ZnIn_2_S_4_ composites. Despite undergoing eight cycles, the composites continue to exhibit elevated levels of photocatalytic hydrogen production without experiencing a notable decline in efficiency. This study introduces a forward-looking methodology for synthesizing and fabricating environmentally sustainable SnO_2_@ZnIn_2_S_4_ composites, which hold promise for applications in the field of photocatalytic hydrogen generation.

## Data Availability

The data presented in this study are available in the article.
